# Effectiveness of Bilateral Arm Training on Upper Limb Functional Mobility in Stroke Survivors

**DOI:** 10.7759/cureus.110927

**Published:** 2026-06-15

**Authors:** Yukti K Agrawal, Suraj Kanase

**Affiliations:** 1 Department of Neurosciences, Krishna College of Physiotherapy, Krishna Vishwa Vidyapeeth (Deemed to be University), Karad, IND; 2 Department of Neurophysiotherapy, Krishna College of Physiotherapy, Krishna Vishwa Vidyapeeth (Deemed to be University), Karad, IND

**Keywords:** hemiparesis, neuroplasticity, post-stroke recovery, task-oriented training, upper extremity rehabilitation

## Abstract

Background: Poor upper limb use resulting from stroke often negatively affects independence and the ability to perform activities of daily living. Furthermore, impairments in motor control and coordination can hinder rehabilitation efforts to recover upper extremity functional mobility after a stroke; hence, the use of bilateral arm training (BAT) may aid in this recovery. BAT aids recovery by promoting interhemispheric communication, decreasing non-use, and promoting symmetry during movement.

Purpose: Our purpose was to investigate the efficacy of BAT for upper extremity functional mobility in stroke survivors.

Method: Twenty-six stroke survivors were sampled using a convenience sampling method in an experimental pre-post design. Participants were divided into two groups (A and B). Participants in Group A engaged in conventional exercise, while those in Group B engaged in BAT. Each group performed four training sessions per week for four weeks; both groups were assessed for upper extremity function using the Fugl-Meyer Assessment of the Upper Extremity (FMA-UE) and Action Research Arm Test (ARAT).

Results: Both Group A and Group B showed significant improvement in Fugl-Meyer Assessment (FMA) and ARAT scores after treatment. However, Group B demonstrated greater improvement than Group A, with highly significant results (p<0.0001).

Conclusion: Overall, both interventions resulted in improved functional mobility of the upper limbs in stroke survivors. However, participants receiving BAT experienced greater improvement than those receiving conventional exercises in both motor recovery and functional performance of their arms. Thus, the intervention provided to Group B (BAT) was determined to be more effective than the intervention provided to Group A (conventional exercises) in improving upper limb function during stroke rehabilitation.

## Introduction

Stroke is a major neurological disorder resulting from an interruption of cerebral blood flow due to ischemia or hemorrhage, leading to temporary or permanent neurological deficits. Stroke continues to be one of the leading causes of mortality and long-term disability worldwide and poses a substantial burden on healthcare systems and rehabilitation services [1-3]. The clinical consequences of stroke include motor, sensory, cognitive, perceptual, and psychological impairments that significantly affect an individual’s functional independence and quality of life [4].

According to the World Stroke Organization, the global burden of stroke has increased considerably over recent decades, with rising incidence, prevalence, and disability-adjusted life years associated with stroke [5,6]. Developing countries such as India contribute significantly to this burden because of the increasing prevalence of hypertension, diabetes mellitus, obesity, smoking, sedentary lifestyles, and other vascular risk factors [7,8]. Epidemiological studies conducted in India have highlighted a growing incidence and prevalence of stroke, making it a major public health concern requiring effective rehabilitation strategies [7,8].

Ischemic and hemorrhagic strokes are the two main types of stroke. Ischemic stroke occurs as a result of a blockage in a cerebral blood vessel, meaning less oxygen and fewer nutrients reach the cells. Hemorrhagic stroke results from the rupture of a blood vessel in the brain, causing bleeding in or around the brain [9-12]. Both forms of stroke produce significant neurological impairments depending on the site and extent of brain injury. Patients may present with hemiplegia, spasticity, impaired balance, altered sensation, speech deficits, cognitive dysfunction, and reduced motor coordination [13].

Among these impairments, upper limb dysfunction is one of the most common and disabling consequences after stroke. Approximately 70-80% of stroke survivors experience upper extremity impairment during the acute phase, and many continue to have persistent deficits even during the chronic stage of recovery [14-16]. Upper limb dysfunction commonly manifests as muscle weakness, abnormal muscle tone, impaired selective motor control, altered proprioception, poor coordination, reduced dexterity, and difficulty performing functional movements [14,17,18]. These impairments can prevent individuals from performing basic everyday tasks such as reaching for objects, grasping objects, dressing, eating, and performing personal hygiene activities. Because of this, a person may be unable to maintain independence or participate fully in social or work-related activities [17,18].

Recovery of upper limb function following stroke remains a major challenge in neurorehabilitation because restoration of hand and arm movements requires highly coordinated neuromuscular control and complex cortical integration [19]. Consequently, rehabilitation interventions targeting upper extremity function have become an essential component of stroke rehabilitation programs. Several therapeutic approaches, including task-oriented training, mirror therapy, virtual reality, robotic-assisted rehabilitation, strengthening exercises, motor relearning strategies, and exergaming, have demonstrated beneficial effects in improving upper limb function after stroke [19-23].

Accurate assessment of upper extremity function is important for evaluating the effectiveness of rehabilitation interventions and monitoring motor recovery. Various standardized outcome measures have been developed for assessing upper limb impairment and functional performance in stroke survivors [24]. The Fugl-Meyer Assessment (FMA) is one of the most widely accepted and validated tools for assessing sensorimotor recovery and motor impairment after stroke [25,26]. Similarly, the Action Research Arm Test (ARAT) is commonly used to evaluate upper limb functional abilities, including grasp, grip, pinch, and gross movement activities [27].

Among the various rehabilitation approaches available for upper limb recovery, bilateral arm training (BAT) has gained considerable attention because of its neurophysiological basis and potential to facilitate motor recovery through simultaneous use of both upper extremities. BAT involves coordinated symmetrical or asymmetrical movement patterns performed simultaneously with both upper limbs [28]. The underlying principle of bilateral training is based on interlimb coupling and activation of both cerebral hemispheres, which may facilitate cortical reorganization and improve motor performance of the affected upper extremity.

Neurophysiological evidence suggests that bilateral movement training may enhance the excitability of the affected motor cortex while reducing transcallosal inhibition from the unaffected hemisphere. This mechanism may promote adaptive neuroplasticity and facilitate recovery of motor function following stroke [28,29]. Repetitive bilateral movement practice may also improve motor learning through enhanced sensory feedback, increased movement repetition, improved timing, and better coordination between the upper limbs.

Previous studies have reported positive effects of bilateral therapy on upper limb function in stroke rehabilitation. Van Delden et al. emphasized the importance of bilateral upper limb training in promoting motor recovery and improving functional performance in stroke survivors [28]. Similarly, Latimer et al., in their systematic review, concluded that bilateral therapy has a significant positive impact on upper extremity function following chronic stroke [29]. These findings suggest that BAT may serve as an effective rehabilitation strategy for improving upper limb functional mobility and motor performance in stroke survivors.

Although previous literature supports the beneficial effects of BAT, limited evidence is available regarding its effectiveness in improving upper limb functional mobility among stroke survivors in the clinical rehabilitation setting. Therefore, the present study was conducted to evaluate the effectiveness of BAT on upper limb functional mobility in stroke survivors.

## Materials and methods

Design and study setting

This experimental pre-post study focused on stroke survivors with impaired upper limb functional mobility. The study was conducted on patients who had experienced a stroke affecting the use of their arms and hands (upper extremities). The Institutional Ethics Committee, Krishna Vishwa Vidyapeeth ("University"), granted ethical approval for this study on November 5, 2025 (KVV/IEC/10/2025 and Protocol #265/2025-2026). Written informed consent was obtained from all participants before enrollment in the study, which was conducted between November 5, 2025, and May 5, 2026.

Participants

Subjects were screened according to predefined inclusion and exclusion criteria, and eligible participants were recruited for the study. Participants included both males and females aged 30-50 years who had experienced a stroke (ischemic or hemorrhagic) at least three months prior to participation and had a Brunnstrom stage of ≥3 with some voluntary movement present in the affected upper limb. Participants were also required to have a Mini-Mental Status Examination (MMSE) [30] score >24, a Fugl-Meyer Assessment (FMA) [25,26] arm section score of 23-47, and the ability to sit unsupported for at least 30 minutes. Participants with severe cognitive impairment, severe spasticity, apraxia, visual or auditory deficits, or any other neurological or musculoskeletal conditions affecting upper limb performance were excluded from the study.

All participants were informed about the purpose and procedures of the study, and written informed consent was obtained prior to participation. A total of 26 stroke survivors were recruited using consecutive sampling and randomly allocated into two groups (n=13 per group) using the chit method. For randomization, 26 identical folded chits were prepared, with 13 chits labeled for the experimental group and 13 for the control group. Each participant was asked to draw one chit from a container without replacement, and group allocation was determined according to the label on the selected chit: Group A: Conventional Group; Group B: Experimental Group.

Procedure

The study was conducted at the Neurosciences OPD of Krishna College of Physiotherapy, Karad, after obtaining institutional ethical approval. Baseline demographic data, including age, sex, and medical history, were collected before the intervention. Pre-intervention assessment of upper limb functional mobility and motor recovery was performed using the FMA-UE [25,26] and the ARAT [27].

The intervention was carried out for approximately 60 minutes, four sessions per week for four weeks. Group A received conventional physiotherapy consisting of upper limb mobility, coordination, and functional task-oriented exercises. The intervention included active-assisted and active range of motion exercises for the shoulder, elbow, wrist, and hand (two sets of 10 repetitions), coordination training involving reaching, grasping, releasing, object manipulation, and pegboard activities (15-20 minutes), and functional task practice such as drinking from a cup, combing hair, buttoning/unbuttoning, and picking up household objects (15-20 minutes). Group B received BAT exercises consisting of symmetrical and coordinated bilateral upper limb activities designed to improve motor recovery, movement coordination, and functional performance. The intervention protocol progressed weekly from simple bilateral reaching and object-handling tasks to more advanced functional and coordination-based activities involving resistance and activities of daily living.

Post-intervention assessment was conducted after completion of the four-week protocol using the same outcome measures. The effectiveness of the interventions was evaluated by comparing pre-test and post-test scores of the FMA and ARAT between the two groups.

A Microsoft Excel (Microsoft® Corp., Redmond, WA, USA) spreadsheet was used for data collection, tabulation, and statistical analysis. Descriptive statistics, including mean and standard deviation (SD), were calculated for all variables of interest. The level of significance was established as p<0.05. GraphPad INSTAT (trial version 3.06, GraphPad Software, Inc., USA) was used to perform the statistical analysis. Prior to statistical analysis, the normality of the data distribution was assessed using the Kolmogorov-Smirnov test. The FMA-UE and ARAT scores were found to be normally distributed (p>0.05). Therefore, parametric tests were deemed appropriate. A paired t-test was conducted to evaluate the difference between pre-intervention and post-intervention scores for each group, while an unpaired t-test was used to compare the relative effectiveness of BAT with traditional arm rehabilitation methods on upper extremity functional mobility in stroke patients. The FMA-UE and ARAT were utilized to assess the recovery of movement and function of the upper extremities.

## Results

Table 1 presents the demographic data of the subjects included in the study. A total of 26 participants were categorized into four age groups: 30-35 years, 36-40 years, 41-45 years, and 46-50 years. The highest number of participants belonged to the 36-40 years age group, comprising nine subjects. This was followed by the 41-45 years age group with eight subjects. The 46-50 years age group included five subjects, while the fewest participants were observed in the 30-35 years age group, with four subjects. The graph indicates that the majority of stroke survivors participating in the study were between 36 and 45 years of age.

**Table 1:** Demographic data FMA, Fugl-Meyer Assessment; ARAT, Action Research Arm Test; SD, standard deviation

Variable	Group A (n=13)	Group B (n=13)	Test used	p-value
Age (years), mean±SD	41.15±5.42	40.92±5.67	Independent t-test	0.91
Gender (male/female)	10 Mar	11 Feb	Chi-square test	1
Stroke type (ischemic/hemorrhagic)	08 May	09 Apr	Chi-square test	0.67
Baseline FMA score	35.38±7.13	35.23±7.09	Independent t-test	0.95
Baseline ARAT score	22.84±6.21	23.07±4.34	Independent t-test	0.91

An independent t-test was performed to compare baseline FMA and ARAT scores between Group A and Group B. No statistically significant differences were observed in baseline FMA scores (p=0.95) or baseline ARAT scores (p=0.91), indicating that both groups were comparable before the intervention. These findings support the success of the randomization procedure. This is shown in Table 2.

**Table 2:** Baseline comparison of outcome measures between the groups FMA, Fugl-Meyer Assessment; ARAT, Action Research Arm Test; SD, standard deviation

Outcome measure	Group A (mean±SD)	Group B (mean±SD)	t-value	p-value	Result
FMA (Pre)	35.38±7.13	35.23±7.09	0.05	0.95	Not significant
ARAT (Pre)	22.84±6.21	23.07±4.34	0.11	0.91	Not significant

Table 3 presents the within-group comparison of FMA and ARAT scores in both Group A and Group B before and after the intervention. For the FMA, Group A showed improvement from a pre-treatment mean of 35.38±7.13 to a post-treatment mean of 46.53±5.33, with a mean difference of 11.15, which was statistically extremely significant (p=0.0001). Group B demonstrated greater improvement from 35.23±7.09 to 59.92±11.65, with a mean difference of 24.69, showing extremely significant results (p<0.0001). For the ARAT, Group A improved from 22.84±6.21 to 30.15±6.23, with a mean difference of 7.30, which was very significant (p=0.0063). Group B showed marked improvement from 23.07±4.34 to 41.61±4.05, with a mean difference of 18.53 and extremely significant results (p<0.0001). Overall, both groups demonstrated improvement following the intervention; however, Group B showed greater improvement compared to Group A in both outcome measures.

**Table 3:** Within-group analysis of outcome measures Statistical analysis was performed using the paired t-test. FMA, Fugl-Meyer Assessment; ARAT, Action Research Arm Test; SD, standard deviation

Outcome measure	Group	Phase	Mean±SD	t-value	p-value	Mean difference	Result
FMA	Group A	Pre	35.38±7.13	4.51	0.0001	11.15	Extremely significant
		Post	46.53±5.33				
	Group B	Pre	35.23±7.09	6.52	<0.0001	24.69	Extremely significant
		Post	59.92±11.65				
ARAT	Group A	Pre	22.84±6.21	2.99	0.0063	7.30	Very significant
		Post	30.15±6.23				
	Group B	Pre	23.07±4.34	11.24	<0.0001	18.53	Extremely significant
		Post	41.61±4.05				

Table 4 presents the between-group comparison of mean differences in FMA and ARAT scores between Group A and Group B after the intervention. For the FMA, Group A showed a mean improvement of 11.15±3.33, whereas Group B demonstrated a greater mean improvement of 24.69±6.72. The difference between the groups was found to be statistically extremely significant (p<0.0001). Similarly, for the ARAT, Group A showed a mean improvement of 7.30±1.97, while Group B showed a greater mean improvement of 18.53±4.11. The comparison between the two groups was also statistically extremely significant (p<0.0001). Overall, the findings indicate that Group B showed significantly greater improvement compared to Group A.

**Table 4:** Between-group analysis of outcome measures Statistical analysis was performed using the independent samples t-test. FMA, Fugl-Meyer Assessment; ARAT, Action Research Arm Test; SD, standard deviation

Outcome measure	Group	N	Mean±SD	Mean difference	t-value	p-value	Result
FMA	Group A	13	11.15±3.33	13.53	6.50	<0.0001	Extremely significant
	Group B	13	24.69±6.72				
ARAT	Group A	13	7.30±1.97	11.23	8.87	<0.0001	Extremely significant
	Group B	13	18.53±4.11				

## Discussion

The present study was conducted to evaluate the effectiveness of BAT on upper limb functional mobility in stroke survivors using the FMA and ARAT as outcome measures. The findings of the study demonstrated statistically significant improvement in upper limb motor recovery and functional performance following the intervention in both groups. However, participants who received BAT demonstrated greater improvement compared to the control group, indicating that BAT is more effective in improving upper extremity functional mobility in stroke survivors.

The significant improvement observed in FMA scores indicates enhancement in motor recovery, voluntary movement control, coordination, reflex activity, and movement synergy of the affected upper extremity following the intervention. Improvement in motor performance may be attributed to repetitive, task-specific, and coordinated bilateral movement patterns practiced during training sessions. BAT promotes simultaneous activation of both cerebral hemispheres and facilitates cortical reorganization through neuroplastic adaptation mechanisms [28,29].

The neurophysiological basis of bilateral training suggests that simultaneous movement of both upper limbs may enhance excitability of the damaged motor cortex while reducing inhibitory influences exerted by the unaffected hemisphere. This interhemispheric facilitation may improve motor recruitment, coordination, and movement execution in the paretic upper limb [28]. Repetitive bilateral movement also provides continuous proprioceptive and sensory feedback, which may further enhance motor relearning and functional recovery.

These findings are also in line with previously published studies that have shown positive effects of using both arms to help improve upper limb function in stroke survivors. According to Latimer et al.'s review, using both arms in therapy helps improve motor recovery and functional performance in individuals who have had a stroke for six months or more [29]. Similarly, van Delden et al. emphasized that bilateral upper extremity training improves movement coordination and promotes functional use of the affected arm during daily activities [28].

Recent systematic reviews and meta-analyses have further strengthened the evidence supporting BAT in stroke rehabilitation. The present study's findings are consistent with those reported by Chen et al., who observed greater improvements in upper extremity motor recovery following BAT compared with conventional therapy. The enhanced outcomes may be attributed to increased activation of both cerebral hemispheres, improved interlimb coordination, and facilitation of neural reorganization, all of which contribute to improved motor performance and functional recovery after stroke [31]. The authors also highlighted that treatment intensity, repetition, and chronicity of stroke influence rehabilitation outcomes following bilateral training interventions.

Similarly, Gnanaprakasam et al. demonstrated that task-based BAT significantly improves upper limb motor recovery and functional performance in stroke survivors [32]. The authors suggested that repetitive bilateral functional activities enhance sensorimotor integration, movement coordination, and motor relearning, thereby contributing to improved upper extremity function.

The significant improvement observed in ARAT scores in the present study reflects enhancement in functional activities involving grasp, grip, pinch, reaching, and object manipulation. Improvement in these components indicates that BAT contributes not only to motor recovery but also to functional task performance required for activities of daily living. The repetitive nature of bilateral training likely improved movement accuracy, timing, coordination, and motor planning necessary for upper limb functional activities [33].

Chen et al. compared bilateral and unilateral upper limb training and reported that bilateral training demonstrates superior effects on motor impairment, coordination, and functional movement performance after stroke [34]. The authors proposed that bilateral symmetrical movement training facilitates motor planning and improves the organization of movement patterns in the affected upper extremity. Similarly, Stewart et al., in their meta-analysis, reported that bilateral movement training significantly improves motor outcomes in stroke rehabilitation by promoting cortical reorganization and enhancing activation of motor pathways [35].

Upper limb dysfunction following stroke significantly affects functional independence and quality of life [14-18]. Therefore, interventions capable of restoring upper extremity function are essential in neurorehabilitation. The present findings support the growing evidence that repetitive and task-oriented rehabilitation approaches play an important role in promoting functional recovery after stroke [19]. BAT incorporates several principles of motor learning, including repetition, task specificity, active participation, sensory feedback, and progressive movement practice, all of which contribute to motor recovery.

The improvement observed in the present study may also be explained by enhanced interlimb coordination achieved during bilateral movement practice. Bilateral symmetrical movements may normalize abnormal movement patterns and facilitate smoother motor execution of the affected limb. Repetitive coordinated movement practice may strengthen neural connections and improve sensorimotor integration within cortical and subcortical motor pathways.

The present findings are clinically important because upper limb dysfunction remains one of the most disabling impairments after stroke, often limiting participation in self-care and functional activities [17,18]. BAT appears to be a simple, cost-effective, and clinically feasible rehabilitation approach that can be easily incorporated into conventional physiotherapy programs for stroke survivors.

The results of this study are also supported by previous rehabilitation research demonstrating the effectiveness of task-oriented interventions such as mirror therapy, virtual reality, robotic rehabilitation, and exergaming in improving upper extremity function after stroke [20-23]. Similar to these approaches, BAT emphasizes active movement participation and repetitive functional practice, which are considered essential for inducing neuroplastic changes and promoting motor recovery.

Although the results of the current study were favorable, several limitations must be acknowledged. The sample size was small, which may limit the external validity of these results. The intervention duration was limited, and outcomes associated with long-term follow-up evaluations were not collected to assess the durability of treatment effects. In addition, the study concentrated primarily on motor and functional outcomes, and no assessment was conducted of psychosocial factors, participation outcomes, or quality of life measures. Another limitation of the present study is that the participants were relatively young stroke survivors (30-50 years of age). Younger individuals generally possess greater neuroplastic potential, fewer comorbidities, and better physical reserve, which may facilitate faster and greater functional recovery. Therefore, the observed treatment effects may overestimate the recovery rate compared to older stroke populations, who represent a larger proportion of typical stroke cohorts.

Future studies should include multicenter trials and extended follow-up periods to further establish the long-term effectiveness of BAT in stroke rehabilitation. Further research may also investigate the combined effects of BAT with advanced neurorehabilitation approaches such as robotic-assisted therapy, virtual reality systems, neuromuscular electrical stimulation, and brain-computer interface technologies to optimize upper limb recovery outcomes in stroke survivors.

## Conclusions

The present study concluded that both conventional exercises and BAT were effective in improving upper limb functional mobility in stroke survivors. Significant improvements were observed in motor recovery and functional arm performance in both groups, as measured using the FMA and ARAT. These findings suggest that repetitive upper limb rehabilitation exercises contribute positively to recovery following stroke.

However, participants who received BAT demonstrated significantly greater improvement compared to those receiving conventional exercises alone. The enhanced outcomes observed in Group B may be attributed to improved interhemispheric coordination, repetitive symmetrical movement practice, and the facilitation of neuroplasticity. Therefore, BAT can be considered a more effective rehabilitation approach for improving upper limb motor function and functional mobility in stroke survivors.
